# Radiological Investigation of High Background Radiation Areas

**DOI:** 10.1038/s41598-017-15201-2

**Published:** 2017-11-09

**Authors:** Fawzia Mubarak, M. Fayez-Hassan, N. A. Mansour, Talaat Salah Ahmed, Abdallah Ali

**Affiliations:** 1 Radiation Protection Dept., Nuclear Research Center, Egyptian Atomic Energy Authority, Cairo, Egypt; 2 Experimental Nuclear Physics Dept., Nuclear Research Center, Egyptian Atomic Energy Authority, Cairo, Egypt; 3 Faculty of Science,Zagazig University, El-Sharkia, Egypt

## Abstract

In this paper, we used the Hyper-Pure Germanium (HPGe) detector to measure 30 samples which are collected from north of Nile Delta near Rosetta beach in Egypt. The activity of primordial radionuclides, such as ^238^U, ^235^U, ^232^Th, and ^40^K was estimated. Concentrations ranged between 36.5–177.4, 50–397.5 and 56.1–168.9 Bq.kg^−1^ for ^238^U, ^232^Th and ^40^K respectively. Activity concentration of ^235^U and the variation in uranium isotopic ratio ^235^U/^238^U was calculated. External hazard indices (H_ex_) (or radium equivalent activity Ra_eq_), activity concentration indices (I), alpha index (I_α_), absorbed outdoor gamma dose rate (D_out_), effective outdoor gamma dose rate (E_out_) and Excess Lifetime Cancer Risk (ELCR) due to different samples are estimated. External hazard indices (H_ex_) are ranged between 0.32–2.04, radium equivalent activity (Ra_eq_) are ranged between 118.67–753.91, the activity concentration indices (I) are 0.42–2.61, and alpha index (I_α_) are 0.18–0.89. External hazard indices (H_ex_) in some samples more than unity then it exceeds the upper limit of exposure. Also, the radium equivalent activities (Ra_eq_) are higher than the exemption limits (370 Bq.kg^−1^).

## Introduction

Human beings always are exposed to natural radiation, which is mainly due to the activity of natural radionuclides: ^238^U (^226^Ra) series, ^232^Th series and ^40^K that are present in the earth’s crust, in building materials, air, water, food and the human body. Naturally occurring radionuclides in soils are the major contributors of outdoor terrestrial natural radiation^1^. Due to these radionuclides are not uniformly distributed, the understanding of their distribution in soil, sand, and rock are very important in radiation protection and measurement^2^. The associated external exposures due to gamma radiation emitted from these radionuclides depend on the geographical and geological conditions and were varied vary from region to another in the world. High background radiation areas, (HBRAs) are distributed through some regions in the world^3^. In Egypt, there are some areas known for their HBRAs whose geological and geochemical characteristics increase the levels of natural radiation. Black sand one of the most famous materials that have high background radiation and it contributes to increasing the environmental dose^4^. Beach sands are mostly composed of feldspar, quartz and other minerals opposing to wave abrasion. They are formed due to fragmentation, weathering, and degradation. Beach placer or “black sand” deposits around Mediterranean Sea’ beaches are known for their economic concentrations of different minerals such as Monazite, Zircon, Biotite, Rutile, Chromite, Garnet, Allanite and Sillimanite, Tourmaline, Sphene, Pyroxenes, Haematite, Ilmenite, Niobian-Rutile, and Pyrrhotite. Pyrrhotite and Niobian-Rutile were found in magnetite and Ilmenite respectively^5,6^. Hinterland geology, sub-tropical climate, geomorphology and intricate network drainage aided by wind, waves, and currents have influenced these formations. Monazite bearing black sands contains ^232^Th with some extent of ^238^U and ^40^K^7,8^. The main activity of the uranium and thorium series is due to the fractions of zircon, Imenite and little of it due to garnet ^238^U activity was controlled by heavy non-magnetic (HNM) fraction (Monazite, Zircon, Titanite and Apatite), while the heavy magnetic (HM) fraction, at least for the heavy mineral rich samples bearing high amounts of Epidote crystals with Allanite cores, control their ^232^Th content^9–11^. Studies concerning the radiation risks arising from exposure to black sand showed that is the main source of external dose to the world population was due to natural radiation^12–14^.

**Figure 1:**
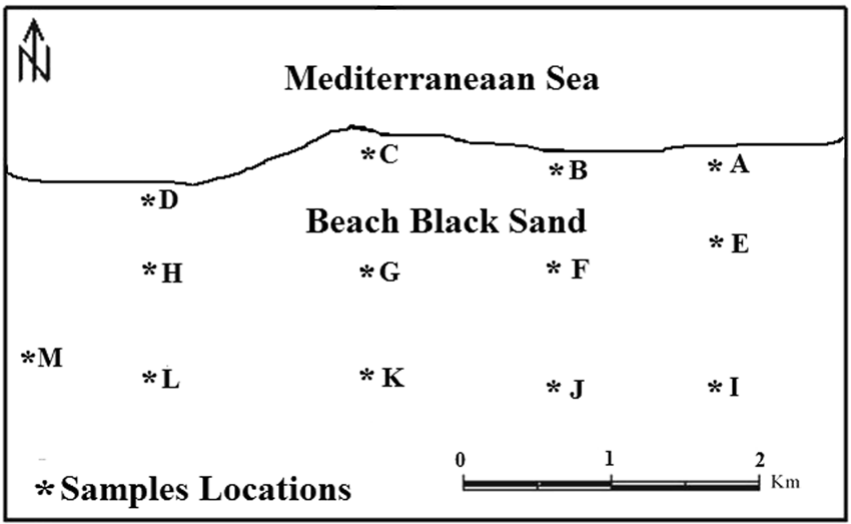
Sampling locations.

This study investigates the distribution of natural radionuclides in the north of Nile Delta near Rosetta beach to understand the radiological risks due to the gamma-ray exposure^15^. Samples were collected from east to west and locations were divided into 12 groups, spaced in-between by about 600 m and extending into the land from the beach line for about 50 m or less, Fig. (1).

## Results

**Figure 2:**
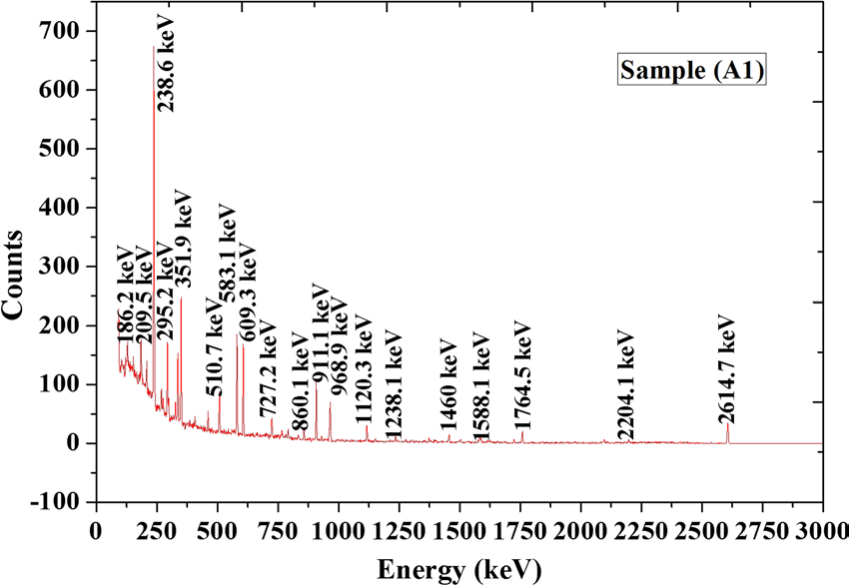
Spectrum analysis for sample (A1).

**Figure 3:**
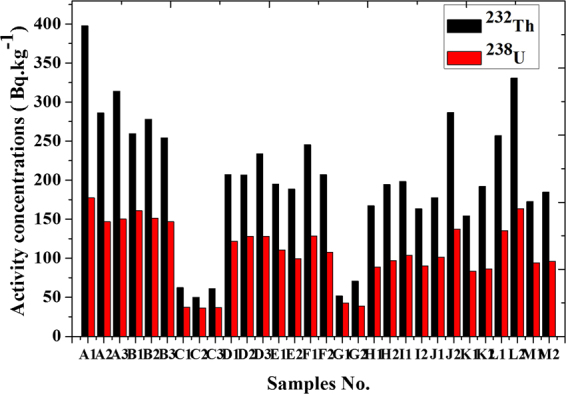
Activity concentrations of ^232^Th and ^238^U (Bq.kg^*−*1^) for different samples.

Figure (2) shows an example of spectrum analysis of black sand beach. Samples are dominated by Th and U-bearing minerals due to the presence of significant amounts of Monazites and Zircon as shown in Fig. (3).

Table (1) shows activity concentration (Bq.kg^−1^) of different samples. They were ranged between 36.5–177.4 with an average of 107.6 Bq.kg^−1^ for ^238^U, 50–397.5 with an average of 201.6 Bq.kg^−1^ for ^232^Th and 56.1–168.9 with an average of 116.2 Bq.kg^−1^ for ^40^K.

**Table 1:** Activity concentration of different samples at different locations and ^235^U/^238^U ratio.

Sample	Activity Concentration (Bq.kg^−1^) ^238^U ^232^Th ^40^K ^235^U				^235^U/^238^U ratio
A1	177.4 ± 39.7	397.5 ± 71.5	105.0 ± 15.8	9.8 ± 8	0.055
A2	146.9 ± 26.6	286.0 ± 12.0	93.0 ± 10.7	7.3 ± 0.5	0.049
A3	150.4 ± 42.9	313.9 ± 12.1	103.0 ± 13.5	6.3 ± 0.4	0.042
B1	160.9 ± 63.9	259.4 ± 50.9	168.9 ± 17.5	11.1 ± 0.9	0.069
B2	151.4 ± 40.7	278.1 ± 36.1	83.5 ± 11.3	9 ± 0.8	0.059
B3	146.7 ± 21.0	254.2 ± 41.0	110.4 ± 12.7	6.6 ± 0.5	0.045
C1	37.6 ± 4.9	62.5 ± 18.3	151.1 ± 14.9	4 ± 0.5	0.106
C2	36.5 ± 7.0	50.0 ± 7.8	138.6 ± 19	2 ± 0.3	0.055
C3	36.6 ± 7.2	61.0 ± 13.8	144.8 ± 17.5	2.5 ± 0.2	0.068
D1	121.6 ± 26.9	207.3 ± 44.6	94.8 ± 11.5	11 ± 08	0.090
D2	128.0 ± 28.1	206.7 ± 20.2	117.6 ± 13.4	5.7 ± 0.6	0.045
D3	128.3 ± 36.2	233.7 ± 51.0	89.4 ± 14.3	9.4 ± 0.8	0.073
E1	110.6 ± 22.7	194.8 ± 24.4	91.8 ± 12.6	6.7 ± 0.7	0.061
E2	99.3 ± 19.8	188.5 ± 32.6	129.9 ± 18.2	5.2 ± 0.5	0.052
F1	128.8 ± 19.0	245.2 ± 36.8	76.0 ± 9.8	10.6 ± 0.9	0.082
F2	107.5 ± 23.5	207.1 ± 34.9	118.0 ± 12.7	7.8 ± 0.7	0.073
G1	42.7 ± 5.4	52.2 ± 13.5	150.0 ± 14.6	3.1 ± 0.2	0.073
G2	39.1 ± 21.4	70.9 ± 5.2	134.1 ± 14.2	3.8 ± 0.2	0.097
H1	88.7 ± 13.7	167.1 ± 39.7	92.9 ± 8.5	5.1 ± 0.4	0.057
H2	96.9 ± 22.7	194.6 ± 13.7	107.5 ± 9.2	11.1 ± 0.9	0.115
I1	103.6 ± 33.5	198.4 ± 32.2	133.8 ± 12.6	3.1 ± 0.2	0.030
I2	90.4 ± 32.0	163.4 ± 38.9	142.2 ± 12.8	6.8 ± 0.5	0.075
J1	101.5 ± 31.8	177.6 ± 21.2	130.5 ± 11.6	5.8 ± 0.4	0.057
J2	137.4 ± 35.6	286.6 ± 61.9	130.7 ± 13.1	10.4 ± 0.8	0.076
K1	83.4 ± 21.6	154.5 ± 25.0	110.1 ± 8.8	2.5 ± 0.2	0.030
K2	86.4 ± 13.8	192.2 ± 9.7	107.5 ± 7.6	5.7 ± 0.3	0.066
L1	135.2 ± 35.7	257.1 ± 47.9	131.8 ± 11.5	7.6 ± 0.7	0.056
L2	163.3 ± 28.1	330.9 ± 65.5	122.8 ± 10.3	10.8 ± 0.9	0.066
M1	93.9 ± 12.5	172.5 ± 34.2	56.1 ± 5.8	8.2 ± 0.6	0.087
M2	95.9 ± 31.3	184.8 ± 30.2	120.5 ± 11.9	2.5 ± 0.4	0.026
Min	36.6 ± 7.2	50.0 ± 7.8	56.1 ± 5.8	2 ± 0.3	0.026
Max	177.4 ± 39.7	397.5 ± 71.5	168.9 ± 17.5	11.1 ± 0.9	0.115
Average ± St. Dev.	107.6 ± 40.2	201.6 ± 84.8	116.2 ± 25.2	6.86 ± 2.9	0.065 ± 0.021

**Figure 4:**
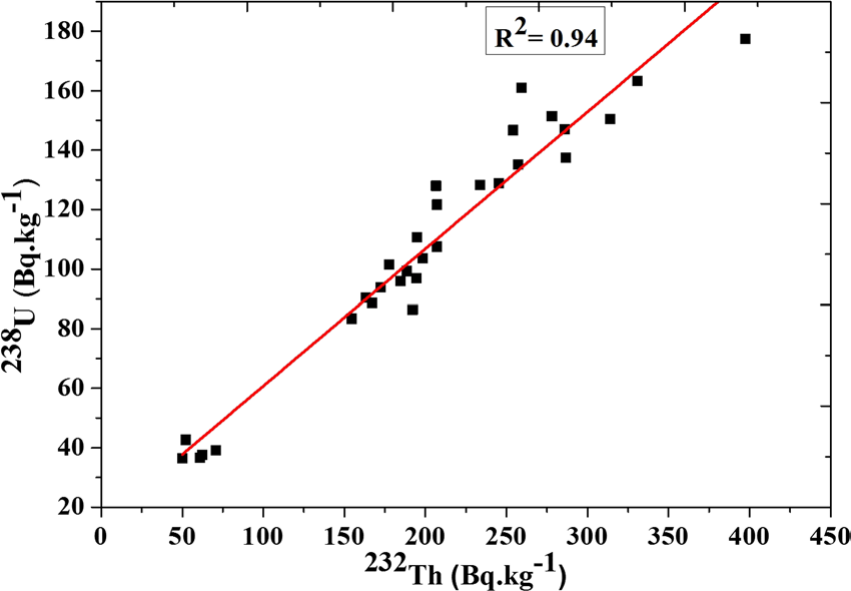
Correlation between the activity concentrations for ^232^Th and ^238^U.

The specific activities ratio (^232^Th/^238^U) in sand samples varied from 1.22 to 2.24 with a mean value of 1.84 this is due to that Monazite contains more thorium than uranium^16^. A correlation exists between the activity of ^232^Th and ^238^U in black sand beach samples (R^2^ = 0.94) with fitting equation ^**232**^ **Th** = **2.17** **×** ^**238**^ **U** **–** **20.71** and standard error ( ± 4.7) as shown in Fig. (4).

**Figure 5:**
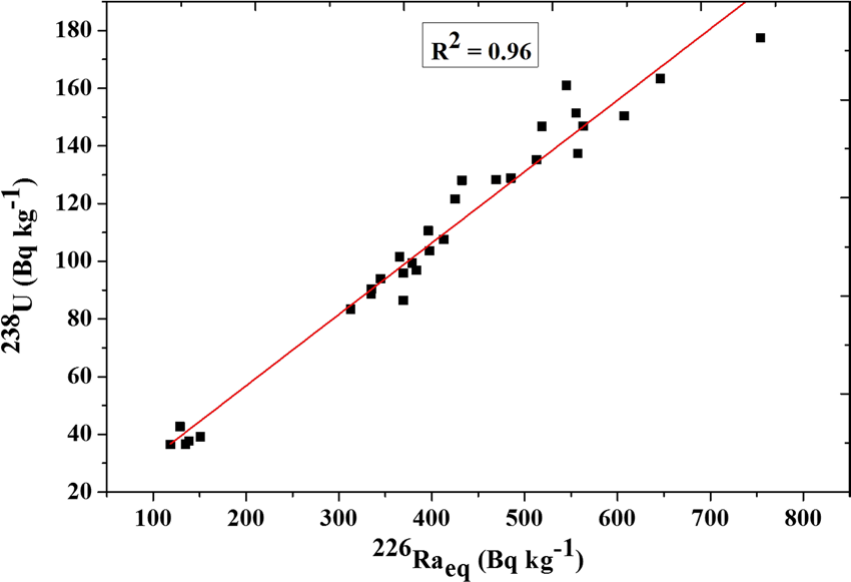
Correlation between the activity concentrations for ^238^U and ^226^Ra_eq_.

Figure (5) illustrates the relation between ^226^Ra_eq_ and ^238^U activity with the fitted straight line (R^2^ = 0.96). This indicated a positive and strong correlation coefficient between uranium concentration and radium equivalent activity level in black sand beach samples.

**Figure 6:**
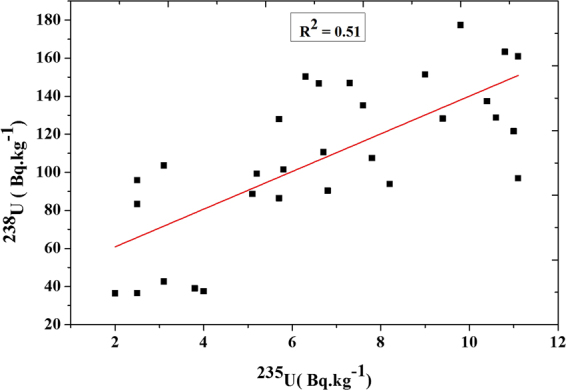
Correlation between the activity concentrations for ^235^U and ^238^U.

**Figure 7:**
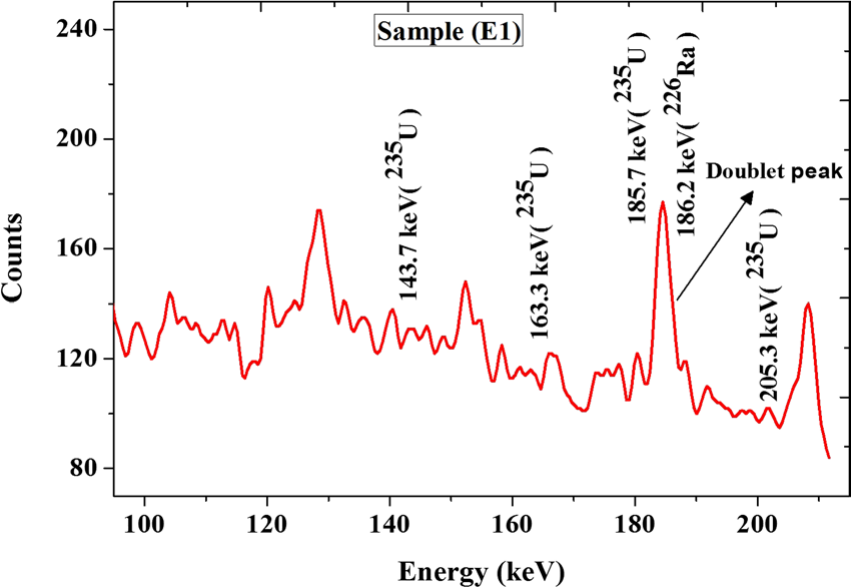
Spectrum of ^235^U Gamma ray energies and doublet peak of ^235^U (185.7 keV) plus ^226^Ra (186.2 keV) for sample (E1).

Activity concentration of ^235^U and the variation in uranium isotopic ratio ^235^U/^238^U also is shown in the Table (1). The specific activities ratio (^235^U/^238^U) in black sand beach samples varied from 0.026 to 0.115 with an average value of 0.065. A correlation exists between ^235^U and ^238^U in black sand beach samples (R^2^ = 0.51) with fitting equation ^**235**^ **U** **=** ^**238**^ **U/9.9** – **4.15** and standard error (±1.1) as shown in Fig. (6). Figure (7) shows an example for U-235 concentration spectrum.

**Figure 8:**
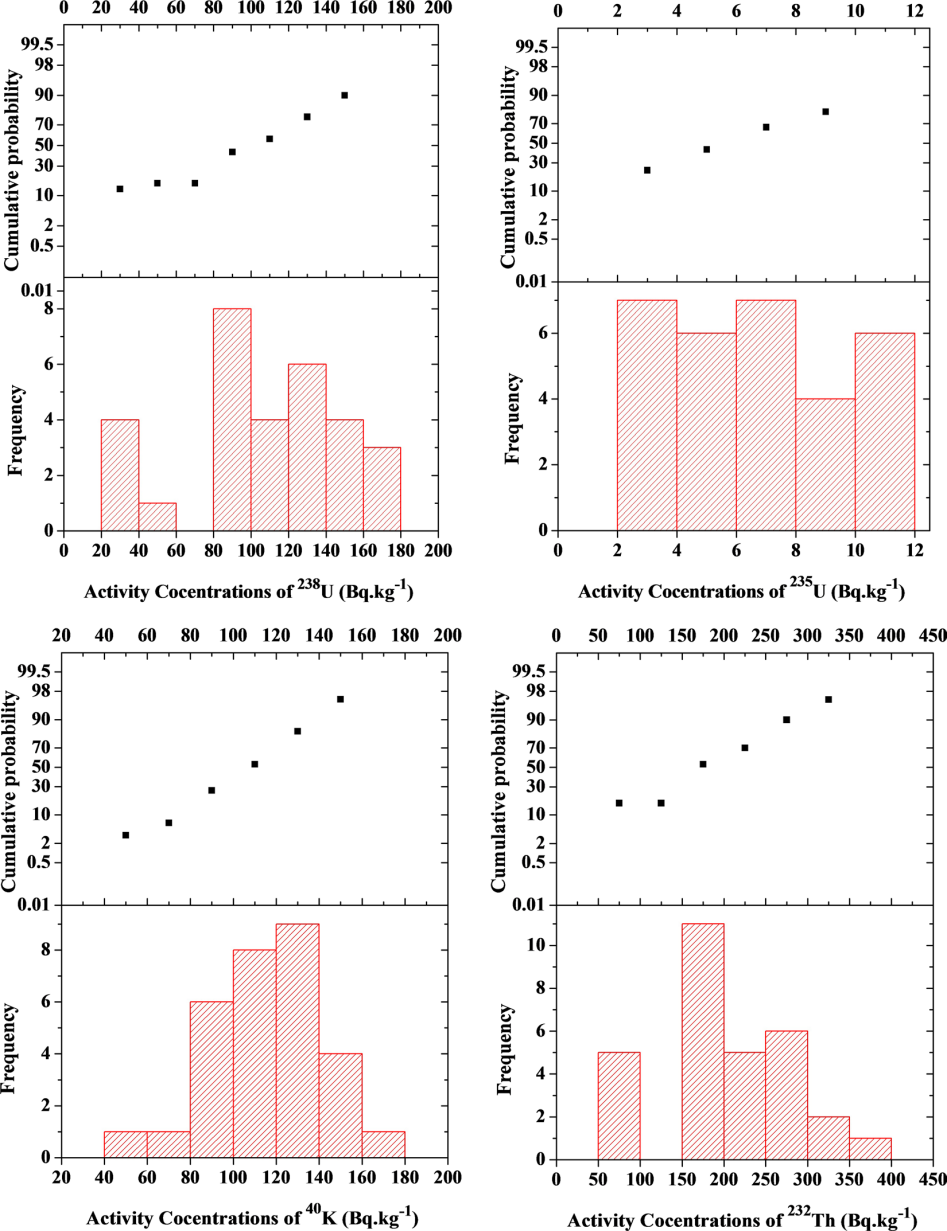
Histogram and cumulative probability plots for the frequency distribution of ^238^U,^235^U,^232^Th and ^40^K for different samples.

The frequency distribution and the cumulative probability of different isotopes (^238^U,^235^U,^232^Th and ^40^K) for different samples are shown in Fig. (8).

Table (2) shows external hazard indices (H_ex_), activity concentration indices (I), radium equivalent activity (Ra_eq_), alpha index (I_α_), absorbed outdoor gamma dose rate (D_out_) and effective outdoor gamma dose rate (E_out_) due to different samples. External hazard indices (H_ex_) were ranged between 0.32 and 2.04, radium equivalent activity (Ra_eq_) were ranged between 118.67 to 753.91, activity concentration indices (I) were 0.42–2.61, and alpha index (I_α_) were 0.18 to 0.89.

**Table 2:** External hazard indices (H_ex_), radium equivalent Ra_eq_, activity concentration indices (I), alpha index I_α_, absorbed dose (D_out_), effective (E_out_) outdoor gamma dose rate and Excessive Lifetime Cancer Risk (ELCR_out_) due to different samples.

_Sample_	_Raeq_	_Hex_	_I_	_Iα_	_Dout_	E_out_	ELCR_out_ × 10^-3^
A1	753.91	2.04	2.61	0.89	345.95	0.42	1.49
A2	563.04	1.52	1.95	0.73	258.01	0.32	1.1
A3	607.21	1.64	2.11	0.75	278.57	0.34	1.20
B1	544.85	1.47	1.89	0.80	249.89	0.31	1.07
B2	555.51	1.50	1.92	0.76	254.24	0.31	1.09
B3	518.71	1.40	1.80	0.73	237.57	0.29	1.02
C1	138.61	0.37	0.49	0.19	64.89	0.08	0.28
C2	118.67	0.32	0.42	0.18	55.51	0.07	0.24
C3	134.98	0.36	0.48	0.18	63.17	0.08	0.27
D1	425.34	1.15	1.47	0.61	194.81	0.24	0.84
D2	432.64	1.17	1.50	0.64	198.23	0.24	0.85
D3	469.37	1.27	1.63	0.64	215.02	0.26	0.92
E1	396.23	1.07	1.37	0.55	181.61	0.22	0.78
E2	378.86	1.02	1.32	0.50	174.35	0.21	0.75
F1	485.29	1.31	1.68	0.64	222.26	0.27	0.95
F2	412.74	1.11	1.43	0.54	189.70	0.23	0.81
G1	128.90	0.35	0.45	0.21	60.18	0.07	0.26
G2	150.81	0.41	0.53	0.20	70.34	0.09	0.30
H1	334.81	0.90	1.16	0.44	153.80	0.19	0.66
H2	383.46	1.04	1.33	0.48	176.32	0.22	0.76
I1	397.61	1.07	1.38	0.52	182.97	0.22	0.79
I2	335.01	0.90	1.17	0.45	154.38	0.19	0.66
J1	365.52	0.99	1.27	0.51	168.06	0.21	0.72
J2	557.30	1.51	1.93	0.69	256.10	0.31	1.10
K1	312.81	0.84	1.09	0.42	143.93	0.18	0.62
K2	369.52	1.00	1.28	0.43	170.21	0.21	0.73
L1	513.00	1.39	1.78	0.68	235.56	0.29	1.01
L2	645.94	1.74	2.24	0.82	296.37	0.36	1.27
M1	344.89	0.93	1.19	0.47	157.90	0.19	0.68
M2	369.44	1.00	1.28	0.48	169.98	0.21	0.73
Min.	118.67	0.32	0.42	0.18	55.51	0.07	0.24
Max.	753.91	2.04	2.61	0.89	345.95	0.42	1.49
Av. ± Stdev	1.1 ± 0.4	404.8 ± 159.9	1.4 ± 0.6	0.54 ± 0.2	186. ± 73	0.2 ± 0.1	0.8 ± 0.3

**Figure 9:**
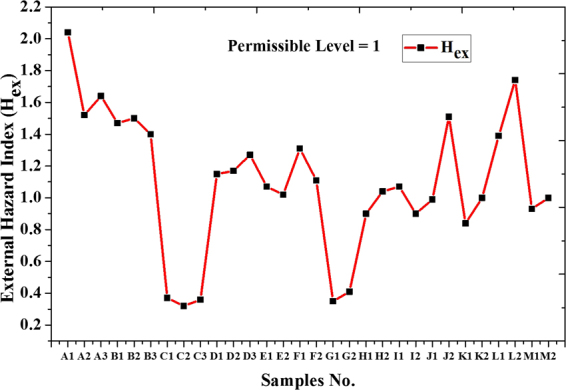
External Hazard Index (H_ex_) for different samples.

**Figure 10:**
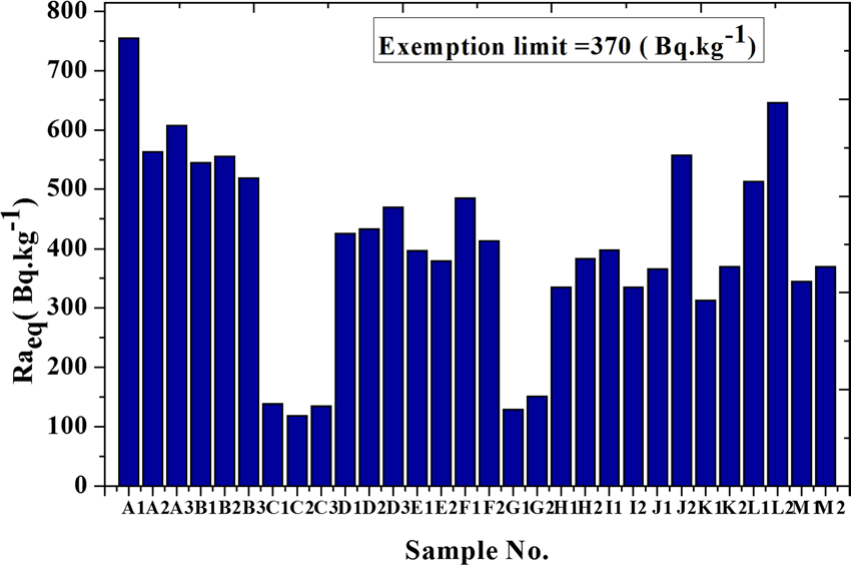
Radium equivalent for different samples.

**Figure 11:**
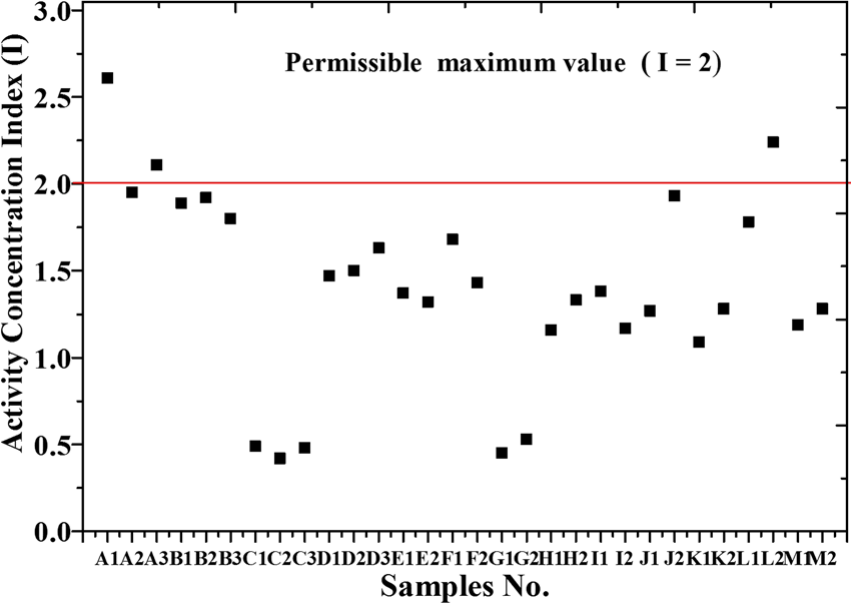
Activity concentration indices for different samples.

These results show that some locations lead to over-exposure, for example, external hazard indices (H_ex_) in some samples are found to be more than unity then it exceeds the upper limit of exposure, Fig. (9). Also, radium equivalent activity (Ra_eq_), Fig. (10), was higher than the exemption limits (370 Bqkg^−1^) that keep the external dose below 1.5 mSvyr^−1^ as reported by UNSCEAR (2010)^17^. While activity concentration indices (I) slightly exceed the permissible limits which met 0.3 mSvyr^−1^ as shown in Fig. (11). Outdoor Excessive Lifetime Cancer Risk (ELCR_out_) is found to be ranged between 0.24E-3-1.49E-3 with an average of 0.8E-3 which is 2.8 times more than the upper limits 0.29E-3^18^.

**Figure 12:**
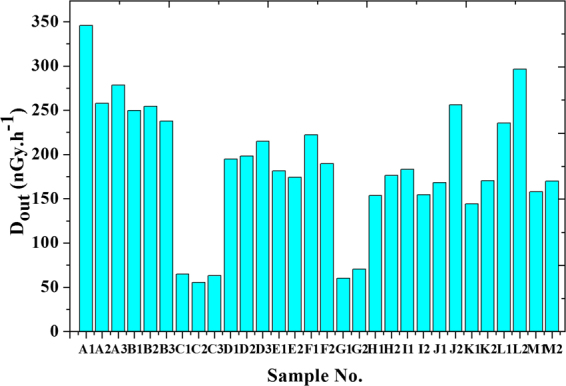
Outdoor absorbed dose for different samples.

Figure (12) shows the outdoor absorbed gamma dose rate (D_out_). It was ranged between 55.51 to 345.95 with an average of 186 nGy.h^−1^ which leads to effective outdoor gamma dose rate (E_out_) ranged between 0.07 to 0.42 with an average of 0.23 mSvyr^−1^ which represented more than 3 times higher than the world’s average of 0.07 mSv.yr^−1^. The outdoor absorbed gamma dose rate was within the range as reported by UNSCEAR-2010 to Nile Delta region which met 20–400 nGy.h^−1^ as shown in the Table (3) in which a comparison between some high background radiation areas among the world was reported.

**Table 3:** A comparison of our study with other studies of black sand beaches^22^.

Country	Area	Area Characteristics	Absorbed dose rate in air (nGy.h^−1^)
Brazil	Guarapari	Monazite sands; coastal areas	90–170 (streets) 90–90000 (beaches) 110–1 300
China	Yangjiang Quangdong	Monazite particles	370 average
India	Kerala Ganges Delta	Monazite sands,	200–4000 1800 average 260–440
Egypt	Nile Delta	Monazite sands	20–400
Present study	North of Nile Delta	Monazite sands	55.51–345.95 186 average

Table (4) shows total annual equivalent dose to different organs and effective dose due to exposure to the average value of the activity of all naturally occurring radionuclides (mSvyr^−1^) in black sand.

**Table 4:** Annual equivalent dose and effective dose due to average activity (mSv.yr^−1^).

Organ	^238^U	^232^Th	^40^K	Summation
R Marrow	3.33 × 10^−8^	5.81 × 10^−7^	6.87 × 10^−4^	6.87 × 10^−4^
Adrenals	2.31 × 10^−8^	5.07 × 10^−7^	7.82 × 10^−4^	7.83 × 10^−4^
B Surface	2.02 × 10^−7^	2.43 × 10^−6^	7.00 × 10^−4^	7.02 × 10^−4^
Brain	2.61 × 10^−8^	5.90 × 10^−7^	6.90 × 10^−4^	6.90 × 10^−4^
Breast	1.62 × 10^−7^	9.62 × 10^−7^	6.72 × 10^−4^	6.73 × 10^−4^
G Bladder	2.21 × 10^−8^	5.01 × 10^−7^	6.32 × 10^−4^	6.33 × 10^−4^
Esophagus	1.60 × 10^−8^	4.44 × 10^−7^	7.39 × 10^−4^	7.40 × 10^−4^
ST Wall	2.99 × 10^−8^	5.87 × 10^−7^	7.34 × 10^−4^	7.35 × 10^−4^
SI Wall	2.03 × 10^−8^	4.90 × 10^−7^	7.26 × 10^−4^	7.27 × 10^−4^
ULI Wall	2.29 × 10^−8^	5.21 × 10^−7^	6.52 × 10^−4^	6.52 × 10^−4^
LLI Wall	2.17 × 10^−8^	5.10 × 10^−7^	6.55 × 10^−4^	6.56 × 10^−4^
Heart	2.73 × 10^−8^	5.64 × 10^−7^	6.67 × 10^−4^	6.67 × 10^−4^
Kidneys	3.54 × 10^−8^	6.07 × 10^−7^	6.85 × 10^−4^	6.86 × 10^−4^
Liver	3.01 × 10^−8^	5.98 × 10^−7^	6.80 × 10^−4^	6.81 × 10^−4^
Lungs	3.57 × 10^−8^	6.73 × 10^−7^	7.31 × 10^−4^	7.32 × 10^−4^
Ovaries	1.99 × 10^−8^	4.72 × 10^−7^	6.49 × 10^−4^	6.49 × 10^−4^
Pancreas	1.86 × 10^−8^	4.78 × 10^−7^	6.27 × 10^−4^	6.28 × 10^−4^
Skin	5.42 × 10^−7^	1.59 × 10^−6^	1.35 × 10^−3^	1.35 × 10^−3^
Spleen	2.93 × 10^−8^	5.98 × 10^−7^	6.87 × 10^−4^	6.87 × 10^−4^
Testes	1.25 × 10^−7^	8.76 × 10^−7^	7.82 × 10^−4^	7.83 × 10^−4^
Thymus	3.62 × 10^−8^	6.41 × 10^−7^	7.00 × 10^−4^	7.00 × 10^−4^
Thyroid	4.45 × 10^−8^	6.44 × 10^−7^	6.90 × 10^−4^	6.91 × 10^−4^
U Bladder	2.90 × 10^−8^	5.67 × 10^−7^	6.72 × 10^−4^	6.72 × 10^−4^
Uterus	1.92 × 10^−8^	4.78 × 10^−7^	6.32 × 10^−4^	6.33 × 10^−4^
Muscle	8.65 × 10^−8^	7.39 × 10^−7^	7.39 × 10^−4^	7.40 × 10^−4^
h_rem	8.07 × 10^−8^	7.22 × 10^−7^	7.34 × 10^−4^	7.35 × 10^−4^
E	6.51 × 10^−8^	6.93 × 10^−7^	7.26 × 10^−4^	7.27 × 10^−4^
Min	1.60 × 10^−8^ (Esophagus)	4.44 × 10^−7^(Esophagus)	6.27 × 10^−4^(Pancreas)	6.28 × 10^−4^(Pancreas)
Max	5.42 × 10^−7^ (Skin)	2.43 × 10^−6^(B-Surface)	1.35 × 10^−3^(Skin)	1.35 × 10^−3^ (Skin)

**Figure 13:**
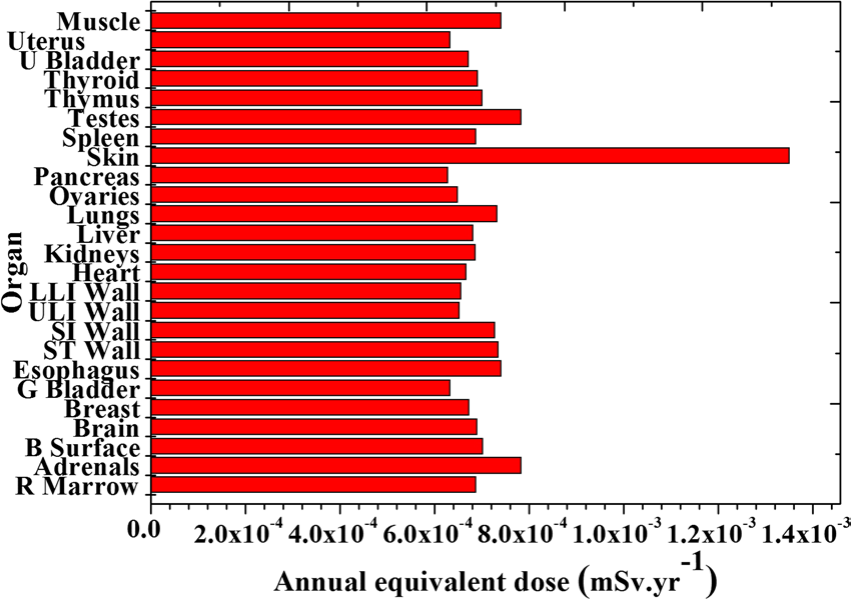
Total equivalent dose to different organs.

Figure (13) investigate the annual equivalent dose to different organs due to the average value of activity concentrations of all-natural radionuclides it was ranged between 6.28E-4 (received by the pancreas) and 1.35E-3 mSv.yr^−1^ (received by skin).

## Discussion

Results show that some locations lead to over-exposure, for example, external hazard indices (H_ex_) in some samples more than unity then it exceeds the upper limit of exposure. Also, radium equivalent activities (Ra_eq_) in the same locations were higher than the exemption limits (370 Bq.kg^−1^). While activity concentration indices (I) slightly exceed the permissible limits which met only 0.3 mSv.yr^−1^. Outdoor absorbed gamma dose rate (D_out_)was ranged between 55.51 to 345.95 with an average of 186 nGy.h^−1^ which leads to effective outdoor gamma dose rate (E_out_) ranged between 0.07 to 0.42 with an average of 0.23 mSvyr^−1^ which represented more than 3 times higher than the world’s average of 0.07 mSvyr^−1^. The outdoor absorbed gamma dose rate was within the range as reported by UNSCEAR-2010 to Nile Delta region which met 20–400 nGy.h^−1^.

In general, it can be concluded that exposure in these areas was still within the permissible limits due to the little time of exposure as these areas are beaches that intended for hiking. Outdoor Excessive Lifetime Cancer Risk (ELCR_out_) is found to be ranged between 0.24 × 10^−3^−1.49 × 10^−3^ with an average of 0.8 × 10^−3^ which is 2.8 times more than the upper limits 0.29 × 10^−3^. It can be noticed that ELCR_out_ in all samples is greater than the upper recommended levels.

Annual equivalent dose due to average concentrations of all-natural radionuclides was ranged between 6.28E-4 (received by the pancreas) and 1.35 × 10^−3^ mSvyr^−1^ (received by skin). Average effective dose due to exposure to all radionuclides was 7.27 × 10^−4^ mSvyr^−1^. It can be concluded that radiological hazard due to external exposure to different organs or tissues were within the international permissible values.

## Material and Methods

About 30 samples were collected from north of Nile Delta near Rosetta beach parallel to the Mediterranean coast. This region is an open area, flat and nearly horizontal^15^. Samples were dried at 105 °C for 12 hours to completely remove residual moisture. About 500 g of each sample was mixed thoroughly, weighed and filled in a polyethylene jar with a screw cover and perfectly sealed with adhesive tapes to make them airtight. These containers were stored for one month at room temperature to allow secular equilibrium between ^226^Ra and its progenies to be achieved before gamma spectroscopy. For Gamma spectrometry a P-type coaxial HPGe detector, Canberra model No., CPVD 30–3020, shielded by 10 cm Pb thickness, 1 mm Cd and 1 mm Cu, with a relative efficiency of 30% and a resolution full width at half maximum (FWHM) of 1.9 keV at 1.33 MeV (with associated electronics) connected to multi-channel analyzer (MCA) and coupled with software program Genie 2000, was used. This detector is of high efficiency and has high resolution, and very low background, that it is important to get an estimate of the detection limits and the minimum detectable activity.

### Efficiency calibration

The efficiency calibration must perform in the same geometry as used in the actual measurements. Efficiency calibration curve was done by using standard calibration sources (powder) produced by IAEA of ^238^U (RGU-1), with 400 ppm concentration, 1.78 density and activity 4.9 Bq/gm and ^232^Th (RGTh-1), with 800 ppm, 1.71 density and activity 3.26 Bq/gm were poured to the similar plastic jar up to the same height as sample. Pure silica was also poured to similar jar up to the same height as a sample. The energy calibration in 63.9–2614 keV was performed using the same standard sources. Determination of NORM was measured using different daughters that emit clear Gamma peaks of high intensity to confirm the attainment of radioactive secular equilibrium within the samples between ^226^Ra and its daughters. This was carried out by measuring ^226^Ra directly through the 186.2 keV and indirectly by measuring the ^214^Bi (609.3, 1120.2 and 1764.5 keV) and ^214^Pb (351.9 keV) photopeaks. ^232^Th was determined through ^228^Ac (911.2 keV) ^212^Pb (238.6 keV after subtracting 241.2 value) and ^208^Tl (2614 keV) photopeaks, and estimation of ^40^K through the 1460.8 keV photopeak. Samples and background were measured for about 6 hours for each.

### Variation in uranium isotopic ratio ^235^U/^238^U

Identification of ^235^U concentration is difficult as its natural abundance concentration is low (only 0.72%) of natural uranium. The energy of 185.7 keV is the most intense gamma-ray line associated with the presence of ^235^U (57%). This is very close to the energy of 186.2 keV associated with the decay of ^226^Ra to ^222^Rn in ^238^U chain. Overlapping may occur during measurement. Due to the relatively lower branching ratios of 143.76 keV (10.96%), 163.33 keV (5.08%) and 205.31 keV (5.01%) energy transitions comparing to that of the 185.7 keV energy transition, they are not commonly used to determine ^235^U in black sand samples. They counting rates due their expected counting rate would be below the detection limits ranges for the HPGe detector. So it is more practical to use the 185.7 keV energy transition to assess the ^235^U. Therefore, the concentration of ^235^U was calculated by subtracting the fraction of ^226^Ra using the following equation,:^19,20^

1 $${}^{235}U=(\frac{\frac{({{\rm{CR}}}_{187}/{\varepsilon }_{{\rm{peak}}})}{{\rm{M}}}-{\rm{AC}}({}^{{\rm{226}}}{\rm{R}}{\rm{a}})\times {{\rm{I}}}_{\gamma }({}^{{\rm{226}}}{\rm{R}}{\rm{a}})}{{{\rm{I}}}_{\gamma }({}^{235}U)})$$

where CR_187_ is the count rate of the peak centered at 187 keV, ε_Peak_ is the detector efficiency at that energy, M is the mass of the sample (kg), I_γ_ (^226^Ra) is the gamma-ray emission fraction for ^226^Ra, I_γ_ (^235^U) is the gamma-ray emission fraction for ^235^U, AC (^226^Ra) is the activity concentration of ^226^Ra in the sample (Bq.kg^−1^) based on the average of the ^214^Bi and ^214^Pb analyses, and ^235^U is the concentration of ^235^U in the sample (Bq.kg^−1^)^21^.

### External and internal hazards calculations

#### External Hazard Index (H_ex_)

It is obtained from Ra_eq_ expression which indicates that the maximum allowable value (equal to unity) corresponds to the upper limit of Ra_eq_ (370 Bq.kg^−1^). This value must be less than unity in order to minimize the radiation hazard, i.e., the radiation exposure must be limited to 1.0 mSv.yr^−1^, then the external hazard index (H_ex_) is given by the following equation:

2 $${H}_{ex}=\frac{{C}_{Ra}}{370}+\frac{{C}_{Th}}{259}+\frac{{C}_{K}}{4810}$$

Where *C*_Ra_, *C*_Th_ and C_K_ are the concentration in (Bq.kg^−1^) of ^226^Ra^232^, Th and ^40^K respectively^22,23^.

#### Radium Equivalent Activity (R_eq_)

External hazard index (H_ex_) can be calculated by another method as expression called radium equivalent activity Ra_eq_ for comparing the specific activity of materials containing different amounts of ^226^Ra^232^, Th and ^40^K. It is based on the fact that 370 (Bqkg^−1^) of ^226^Ra, 259 (Bqkg^−1^) of ^232^Th and 4810 (Bqkg^−1^) of ^40^K, produce the same γ-ray dose equivalent. It is defined by the following expression^17,24^:

3 $$R{a}_{eq}={C}_{Ra}+1.43{C}_{Th}+0.077{C}_{K}$$

### Activity Concentration Index (I)

The activity concentration index should be used for identifying materials which might be of concern. It is used to present investigation levels in the form of an activity concentration index, (I), or shortly, gamma index (I), and it is defined as follows^25^:

4 $$I=\frac{{C}_{Ra}}{300}+\frac{{C}_{Th}}{200}+\frac{{C}_{K}}{3000}$$

The maximum value for activity concentration index is 2 (I ≤ 2) to meet 0.3 mSvyr^−1^dose criterion and I ≤ 6 to meet 1 mSvyr^−1^ ^26,27^.

**Alpha Index (I**_**α**_ **):**

The alpha index is used to assess the excess alpha radiation internal exposure caused by inhalation of naturally occurring radionuclides. When the activity concentration of ^226^Ra exceeds a value of 200 Bqkg^−1^, it is possible that the radon exhaled from this material met a concentration of 200 Bqm^−3^. Many countries of the world suggested the exemption level and upper level of ^226^Ra activity of 100Bqkg^−1^ and 200 Bqkg^−1^ respectively^28^.

**Outdoor Absorbed Gamma Dose Rate (D**_**out**_ **):**

The outdoor absorbed gamma dose rate (D_out_) at 1 meter above the ground surface due to uniformly distributed natural radionuclides can be calculated as follows^29,30^:

5 $${D}_{out}=0.43\,{C}_{Ra}+0.666\,\,{C}_{Th}+0.047\,{C}_{K}(nGy.{h}^{-1})$$

**Annual Outdoor Effective Dose (E**_**out**_ **):**

Annual outdoor effective dose (E_out_) can be calculated from the dose rate (D_out_), about 20% of 8760 hours in a year can be considered as time of stay in the outdoor and for converting absorbed dose in air to effective dose a factor of 0.7 SvGy^−1^ was used, and can be calculated as follow^31^:

6 $${E}_{out}(\frac{mSv}{y})={D}_{out}(\frac{nGy}{h})\times o.7\times 8766\times 0.2\times {10}^{-6}$$

**Excessive Lifetime Cancer Risk (ELCR):**

Outdoor Excess Lifetime Cancer Risk (ELCR_out_) is calculated from outdoor annual effective dose according to the following equation^18^:

7 $$ELC{R}_{out}={E}_{out}\times LE\times RF\,$$

Where LE represents the life expectancy (70 years) and RF represents the fatal risk factor per Sievert (Sv^−1^). ICRP-60^32^ uses RF values of 0.05 for the public in case of stochastic effects.

**External Equivalent and Effective Dose to Organs or Tissues:**

Organ doses due to external exposure were determined by Eckerman and Ryman (DFEXT-code)^33^. The coefficients in this software represent the dose per unit integrated exposure or the dose rate per unit concentration (Sv m^3^/sec Bq). So, activity concentrations were transformed from Bqkg^−1^ into Bqm^−3^.

Where the summation extends over the organs/tissues with explicit W_t_, W_rem_ is the weighting factors for the remainder (0.2), and h_rem_ is the committed dose equivalent per unit integrated exposure for the remainder tissues. h_rem_ is given as:h_rem_ = 1/5 ∑ h_t_.

From these coefficients, equivalent dose (H_t_) to any organ from any radionuclide can be calculated as follows:

8 $${{\rm{H}}}_{{\rm{T}}}={\rm{C}}\times {\rm{T}}\times 3600(\sec \,/\mathrm{hr})\times {{\rm{h}}}_{{\rm{t}}}({\rm{Sv}})$$

While the effective dose (E) can be calculated as follows:

9 $${\rm{E}}={\rm{C}}\times {\rm{T}}\times 3600\times {\rm{e}}({\rm{Sv}})$$

Where:

-h_t_ is the equNivalent dose in tissue (t) per unit integrated exposure (Sv m^3^/sec Bq),
-e is the effective dose per unit integrated exposure = ∑W_t_h_t_, using W_t_ from ICRP-60,
-C is the activity concentration in black sand (Bq/m^3^),
-T is the exposure time (8766 × 0.2 h/year).
