# Author Correction: Radiological Investigation of High Background Radiation Areas

**DOI:** 10.1038/s41598-020-63287-y

**Published:** 2020-04-15

**Authors:** Fawzia Mubarak, M. Fayez-Hassan, N. A. Mansour, Talaat Salah Ahmed, Abdallah Ali

**Affiliations:** 1 Radiation Protection Dept., Nuclear Research Center, Egyptian Atomic Energy Authority, Cairo, Egypt; 2 Experimental Nuclear Physics Dept., Nuclear Research Center, Egyptian Atomic Energy Authority, Cairo, Egypt; 3 Faculty of Science, Zagazig University, El-Sharkia, Egypt

Correction to: *Scientific Reports* 10.1038/s41598-017-15201-2, published online 09 November 2017

This Article contains a typographical error in the title of Table 3.

“A comparison of our study with other studies of black sand beaches^22^.”

should read:

“A comparison of our study with other studies of black sand beaches^17^.

In addition, Reference 17 contains a typographical error in the year of publication. The correct Reference 17 appears below as Reference 1.
